# Morphology and Phylogeny of *Gnomoniopsis* (*Gnomoniaceae*, *Diaporthales*) from *Fagaceae* Leaves in China

**DOI:** 10.3390/jof7100792

**Published:** 2021-09-24

**Authors:** Ning Jiang, Hermann Voglmayr, Dan-Ran Bian, Chun-Gen Piao, Sheng-Kun Wang, Yong Li

**Affiliations:** 1 Key Laboratory of Forest Protection of National Forestry and Grassland Administration, Research Institute of Forest Ecology, Environment and Protection, Chinese Academy of Forestry, Beijing 100091, China; n.jiang@caf.ac.cn (N.J.); bdr@caf.ac.cn (D.-R.B.); cfcc@caf.ac.cn (C.-G.P.); 2 Department of Botany and Biodiversity Research, University of Vienna, Rennweg 14, A-1030 Vienna, Austria; hermann.voglmayr@univie.ac.at; 3 Research Institute of Tropical Forestry, Chinese Academy of Forestry, Guangzhou 510520, China; skwang@caf.ac.cn

**Keywords:** *Ascomycota*, leaf disease, new species, oak, taxonomy

## Abstract

*Gnomoniopsis* (*Gnomoniaceae*, *Diaporthales*) is a well-classified genus inhabiting leaves, branches and fruits of the hosts in three plant families, namely *Fagaceae*, *Onagraceae* and *Rosaceae*. In the present study, eighteen *Gnomoniopsis* isolates were obtained from diseased leaves of *Fagaceae* hosts collected from Fujian, Guangdong, Hainan, Henan, Jiangxi and Shaanxi provinces in China. Morphology from the cultures and phylogeny based on the 5.8S nuclear ribosomal DNA gene with the two flanking internally transcribed spacer (ITS) regions, the translation elongation factor 1-alpha (*tef1*) and the beta-tubulin (*tub2*) genes were employed to identify these isolates. As a result, seven species were revealed, viz. *Gnomoniopsis* *castanopsidis*, *G.* *fagacearum*, *G*. *guangdongensis*, *G.* *hainanensis*, *G*. *rossmaniae* and *G*. *silvicola* spp. nov, as well as a known species *G. daii*. In addition, *G. daii* was firstly reported on the host *Quercus* *aliena*.

## 1. Introduction

*Diaporthales* is a species-rich fungal order usually associated with forest trees as endophytes, pathogens and saprophytes [1,2,3,4,5,6,7,8,9,10]. Amongst the numerous tree pathogens, the most notorious one is *Cryphonectria parasitica* (*Cryphonectriaceae*) causing chestnut (*Castanea* spp.) blight worldwide [11,12,13]. An example for endophytic lifestyle is *Diaporthe biconispora* (*Diaporthaceae*) and an additional six *Diaporthe* species that are endophytic in healthy *Citrus* tissues in China [14]. As an example of a saprophyte, *Apiosporopsis carpinea* (*Apiosporopsidaceae*) occurs on over-wintered leaves of *Carpinus betulus* [15].

*Gnomoniaceae* is a large family of the *Diaporthales*, with currently 38 accepted genera, including *Gnomoniopsis* [16,17,18,19]. *Gnomoniopsis*, based on the type species *G. chamaemori*, is a well-studied genus in regard to morphology, phylogeny and host associations. This genus is characterized by having small, black perithecia immersed in the host tissue and one-septate, oval to fusiform ascospores, and is well-distinguished by phylogenies based on the 5.8S nuclear ribosomal DNA gene with the two flanking internally transcribed spacer (ITS) regions, the translation elongation factor 1-alpha (*tef1*) and the beta-tubulin (*tub2*) genes [20,21]. Species of *Gnomoniopsis* are currently known to inhabit only members of three plant families as hosts, viz. *Fagaceae*, *Onagraceae* and *Rosaceae* [20,21,22,23,24].

Until now, thirty species epithets of *Gnomoniopsis* have been recorded in Index Fungorum, six of them were reported from fagaceous trees [22]. Two species, *Gnomoniopsis clavulata* and *G. paraclavulata*, were firstly discovered on overwintered leaves of *Quercus* trees in the USA [20,21]. Subsequently, *Gnomoniopsis smithogilvyi* with its synonym *G. castaneae* were proposed from rotten fruits of *Castanea* in Australia and Europe by two independent studies [25,26]. However, these two names were proven to be a single species based on phylogeny and morphological characters [27]. Hence, *G. castaneae* becomes a synonym of *G. smithogilvyi* based on priority. In China, *G. daii* was described from rotten fruits and diseased leaves of *Castanea mollissima* [23,28]. Meanwhile, a different species named *G. chinensis* was reported to cause Chinese chestnut branch canker [29]. Later, Yang et al. described *G. xunwuensis* from leaf spots of *Castanopsis fissa* in China [24]. Since three *Fagaceae*-inhabiting species from China are now only known in the asexual morph, it is hard to separate them based on only morphological characters [23,24,29]. Hence, it is necessary to conduct phylogenetic analyses in order to recognize and identify the species [29].

*Fagaceae* is a common plant family widely distributed in the northern hemisphere, with seven genera namely *Castanea*, *Castanopsis*, *Cyclobalanopsis*, *Fagus*, *Lithocarpus*, *Quercus* and *Trigonobalanus* [30]. Previously, *Gnomoniopsis* has been reported from *Castanea*, *Castanopsis* and *Quercus* species [22]. The aims of present study are to investigate fagaceous hosts to collect *Gnomoniopsis* samples in China, and to identify them to species level based on combined morphology and phylogeny of ITS, *tef1* and *tub2* loci.

## 2. Materials and Methods

### 2.1. Field Sampling and Isolation

In the present study, we investigated leaf diseases of fagaceous trees in Fujian, Guangdong, Hainan, Henan, Jiangxi and Shaanxi provinces of China during 2018 and 2020. The diseased leaf samples were packed in paper bags and transferred to the laboratory for isolation. The infected leaves were firstly surface-sterilized for 1 min in 75% ethanol, 3 min in 1.25% sodium hypochlorite, and 1 min in 75% ethanol, then rinsed for 2 min in distilled water and blotted on dry sterile filter paper. Then samples were cut into 0.5 × 0.5 cm pieces using a double-edge blade, and transferred onto the surface of potato dextrose agar (PDA; 200 g potatoes, 20 g dextrose, 20 g agar per L) and malt extract agar (MEA; 30 g malt extract, 5 g mycological peptone, 15 g agar per L), and incubated at 25 °C to obtain the pure culture. The cultures were deposited in China Forestry Culture Collection Center (CFCC), and the specimens in the herbarium of the Chinese Academy of Forestry (CAF).

### 2.2. DNA Extraction, Sequencing and Phylogenetic Analyses

Genomic DNA was extracted from mycelia grown on cellophane-covered PDA using a cetyltrimethylammonium bromide (CTAB) method [31]. DNA was checked by electrophoresis in 1% agarose gel, and the quality and quantity were measured using a NanoDrop 2000 (Thermo Scientific, Waltham, MA, USA). Three partial loci, ITS region, *tef1* and *tub2* genes were amplified by the following primer pairs: ITS1 and ITS4 for ITS [32], EF1-688F and EF2 for *tef1* [33], and T1/Bt2a and Bt2b for *tub2* [34,35]. The polymerase chain reaction (PCR) conditions were as follows: an initial denaturation step of 5 min at 94 °C, followed by 35 cycles of 30 s at 94 °C, 50 s at 48 °C (ITS) or 54 °C (*tub2*) or 55 °C (*tef1*), and 1 min at 72 °C, and a final elongation step of 10 min at 72 °C. PCR products were assayed via electrophoresis in 2% agarose gels. DNA sequencing was performed using an ABI PRISM 3730XL DNA Analyser with a BigDye Terminator Kit v.3.1 (Invitrogen, Waltham, MA, USA) at the Shanghai Invitrogen Biological Technology Company Limited (Beijing, China).

The sequences obtained in the present study were assembled using SeqMan v.7.1.0, and reference sequences were retrieved from the National Center for Biotechnology Information (NCBI), based on recent publications on the genus *Gnomoniopsis* [20,21,22,23,24,29]. Sequences of an accession of *Apiognomonia errabunda* (AR 2813) were added to represent the outgroup. The sequences were aligned using MAFFT v.6 and corrected manually using MEGA 7.0.21 [36].

The phylogenetic analyses of the ITS region and of a combined matrix of the three loci (ITS-*tef1*-*tub2*) were performed using Maximum Likelihood (ML) and Bayesian Inference (BI) methods. ML was implemented on the CIPRES Science Gateway portal (https://www.phylo.org) using RAxML-HPC BlackBox 8.2.10 [37,38], employing a GTRGAMMA substitution model with 1000 bootstrap replicates. Bayesian inference was performed using a Markov Chain Monte Carlo (MCMC) algorithm in MrBayes v. 3.0 [39]. Two MCMC chains, starting from random trees for 1,000,000 generations and trees, were sampled every 100th generation, resulting in a total of 10,000 trees. The first 25% of trees were discarded as burn-in of each analysis. Branches with significant Bayesian Posterior Probabilities (BPP > 0.9) were estimated in the remaining 7500 trees. Phylogenetic trees were viewed with FigTree v.1.3.1 and processed by Adobe Illustrator CS5. The nucleotide sequence data of the new taxa were deposited in GenBank, and the GenBank accession numbers of all accessions included in the phylogenetic analyses are listed in Table 1.

### 2.3. Morphological Identification and Characterization

The morphological data of the isolates collected in the present study were based on the cultures sporulating on PDA in the dark at 25 °C. The conidiomata were observed and photographed under a dissecting microscope (M205 C, Leica, Wetzlar, Germany). The conidiogenous cells and conidia were immersed in tap water, then the microscopic photographs were captured with an Axio Imager 2 microscope (Zeiss, Oberkochen, Germany) equipped with an Axiocam 506 color camera, using differential interference contrast (DIC) illumination. More than 50 conidia were randomly selected for measurement. Culture characteristics were recorded from PDA and MEA after 10 days incubation at 25 °C in the dark.

## 3. Results

### 3.1. Phylogeny

**Figure 1:**
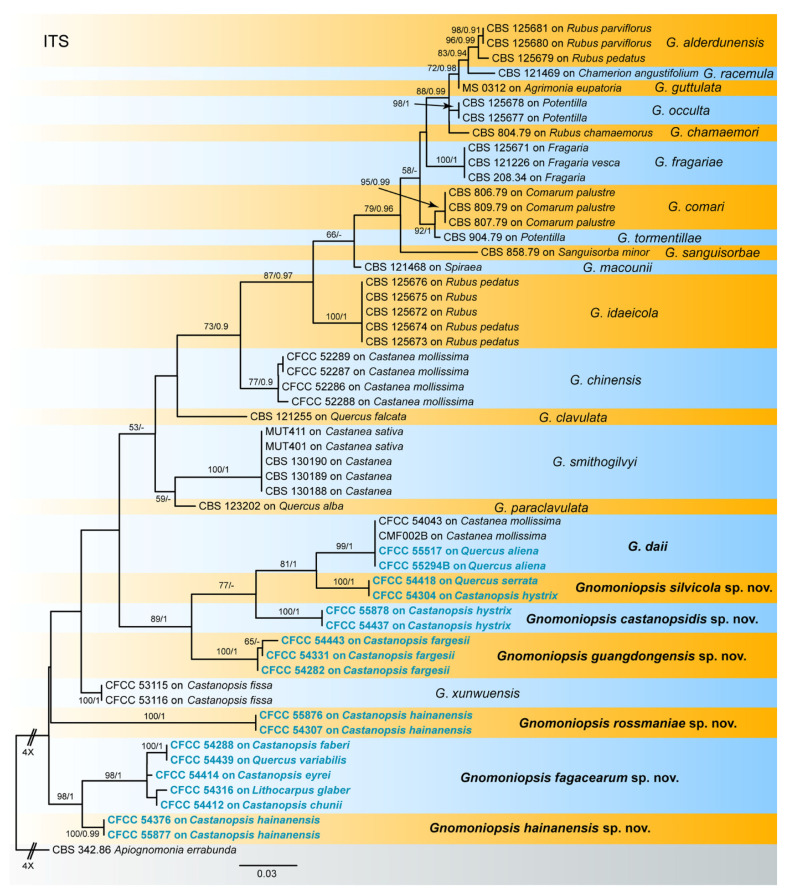
Phylogram of *Gnomoniopsis* resulting from a maximum likelihood analysis based on the ITS gene. Numbers above the branches indicate ML bootstrap values (left, ML BS ≥ 50%) and Bayesian Posterior Probabilities (right, BPP ≥ 0.9). The tree is rooted with *Apiognomonia errabunda* (CBS 342.86). Isolates from the present study are marked in blue, and taxa in bold face are studied in the present study.

The sequence dataset of the ITS gene matrix was analysed to infer the interspecific relationships within *Gnomoniopsis*. The dataset consisted of 56 sequences including one outgroup taxon, *Apiognomonia errabunda* (CBS 342.86). A total of 538 characters including gaps were included in the phylogenetic analysis. The topologies resulting from ML and BI analyses of the concatenated dataset were congruent (Figure 1). Isolates from the present study formed seven individual clades representing seven species of *Gnomoniopsis*, including six new species and one known species.

**Figure 2:**
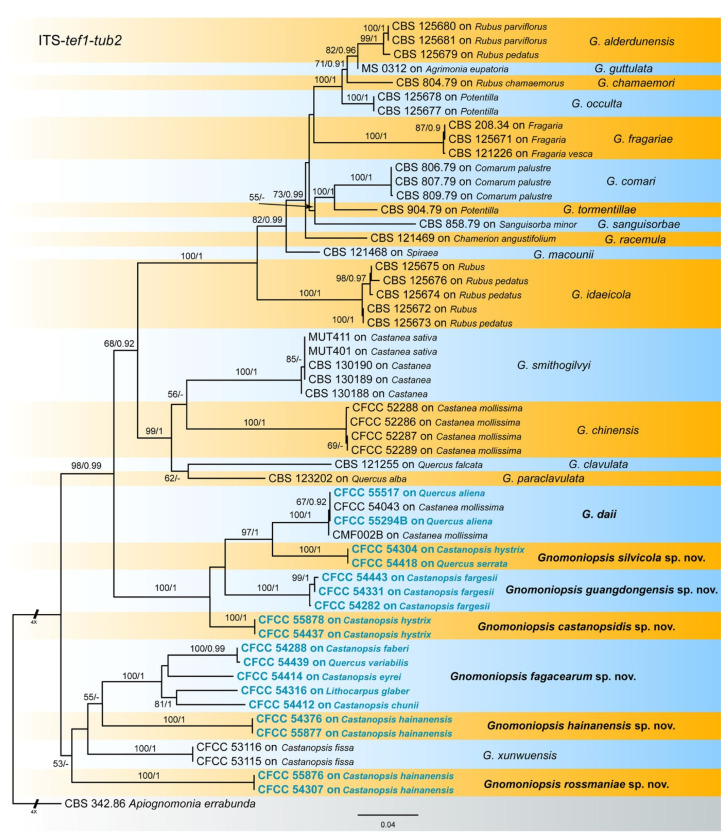
Phylogram of *Gnomoniopsis* resulting from a maximum likelihood analysis based on a combined matrix of ITS, *tef1* and *tub2*. Numbers above the branches indicate ML bootstrap values (left, ML BS ≥ 50%) and Bayesian Posterior Probabilities (right, BPP ≥ 0.9). The tree is rooted with *Apiognomonia errabunda* (CBS 342.86). Isolates from present study are marked in blue, and taxa in bold face are studied in the present study.

The combined three-gene sequence dataset (ITS, *tef1* and *tub2*) was further analysed to compare with results of the phylogenetic analyses of the ITS gene. The dataset consisted of 56 sequences including one outgroup taxon, *Apiognomonia errabunda* (CBS 342.86). A total of 1426 characters including gaps (538 for ITS, 348 for *tef1* and 540 for *tub2*) were included in the phylogenetic analysis. The topologies resulting from ML and BI analyses of the concatenated combined dataset were congruent (Figure 2). Isolates from the present study formed seven individual clades which were congruent with those in Figure 1.

### 3.2. Taxonomy

**Figure 3:**
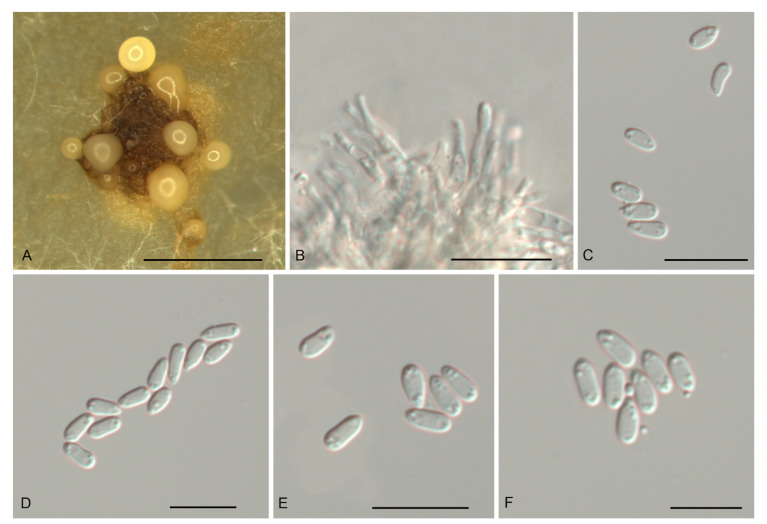
Morphology of *Gnomoniopsis castanopsidis* (CFCC 54437). (**A**) Conidiomata formed on PDA; (**B**) Conidiogenous cells giving rise to conidia; (**C**–**F**) Conidia. Scale bars: A = 500 μm; (**B**–**F**) = 10 μm.

*Gnomoniopsis castanopsidis* N. Jiang, sp. nov. Figure 3.

Mycobank No.: 840969.

Etymology—Named after the host genus, *Castanopsis*.

Description—*Conidiomata* pycnidial, aggregated or solitary, erumpent, globose to pulvinate, brown, 300–700 μm diam., exuding a creamy conidial mass. *Conidiophores* indistinct, often reduced to conidiogenous cells. *Conidiogenous cells* hyaline, smooth, multi-guttulate, cylindrical to ampulliform, attenuate towards apex, phialidic, 6.5–13 × 1.5–3 μm. *Conidia* aseptate, hyaline, smooth, multi-guttulate, oval to fusoid, straight or slightly curved, base truncate, (4.3–) 4.6–5.1 (–5.4) × (1.8–) 2.1–2.5 (–2.6) μm (*n* = 50), L/W = 1.8–2.6.

Culture characteristics—Colonies flat, spreading, with moderate aerial mycelium and undulate margin, fawn on MEA, dirty-white to fawn on PDA, forming abundant brown conidiomata with creamy conidial masses.

Material examined—CHINA, Hainan Province, Changjiang Li Autonomous County, on diseased leaves of *Castanopsis hystrix*, 16 November 2018, Yong Li (JNH0003 *holotype*; *ex-type living culture*, CFCC 54437); *Ibid*. (living culture CFCC 55878).

Notes—Two isolates from leaf spots of *Castanopsis hystrix* clustered into a well-supported clade named *Gnomoniopsis castanopsidis*, which is distinct from any known species phylogenetically (Figure 1 and Figure 2). Morphologically, *G. castanopsidis* is similar to *G. silvicola* in conidial size and shape. However, *G. castanopsidis* is separated from *G. silvicola* in 36 bp differences in ITS.

**Figure 4:**
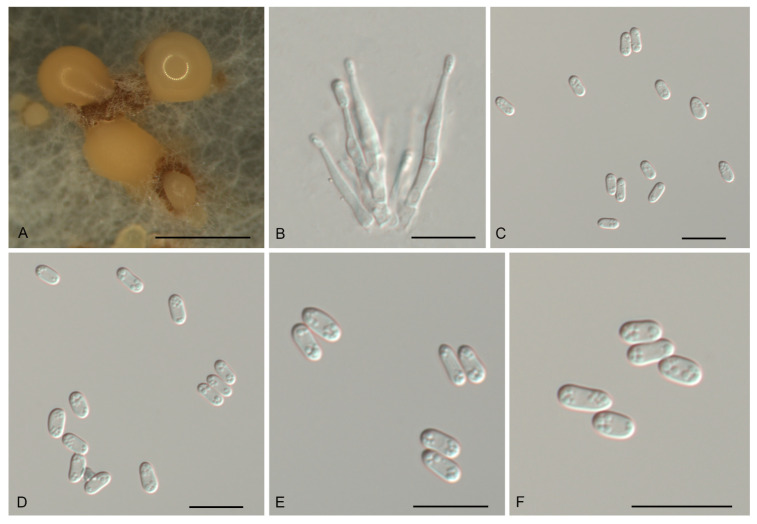
Morphology of *Gnomoniopsis daii* (CFCC 55517). (**A**) Conidiomata formed on PDA; (**B**) Conidiogenous cells giving rise to conidia; (**C**–**F**) Conidia. Scale bars: A = 500 μm; (**B**–**F**) = 10 μm.

*Gnomoniopsis daii* C.M. Tian & N. Jiang, Forests 10(11/1016): 6 (2019). Figure 4.

Description—*Conidiomata* pycnidial, aggregated or solitary, erumpent, globose to pulvinate, brown, 200–600 μm diam., exuding a creamy conidial mass. *Conidiophores* indistinct, often reduced to conidiogenous cells. *Conidiogenous cells* hyaline, smooth, multi-guttulate, cylindrical, attenuate towards apex, phialidic, 7.5–19.5 × 2–3.5 μm. *Conidia* aseptate, hyaline, smooth, multi-guttulate, oval to fusoid, straight or slightly curved, base truncate, (5.1–) 5.6–6.1 (–6.3) × (2.3–) 2.8–3.2 (–3.6) μm (*n* = 50), L/W = 1.4–2.5.

Culture characteristics—Colonies flat, spreading, with moderate aerial mycelium and undulate margin, dirty-white to sienna on MEA, dirty-white to fawn on PDA, forming abundant brown conidiomata with creamy conidial masses.

Material examined—CHINA, Henan Province, Xinyang City, Shihe District, on diseased leaves of *Quercus aliena*, 7 August 2019, Yong Li (JNH0004; living culture, CFCC 55517); *Ibid*. (living culture CFCC 55294B).

Notes—*Gnomoniopsis daii* was initially described as the pathogen of Chinese chestnut (*Castanea mollissima*) fruit rot [23], and subsequently discovered to be the leaf spot pathogen of Chinese chestnut [28]. In the present study, two isolates from diseased leaves of *Quercus aliena* formed a well-supported clade with the ex-type strain of *G. daii* (Figure 1 and Figure 2). Hence, *Gnomoniopsis daii* is for the first time reported on the host genus *Quercus*.

**Figure 5:**
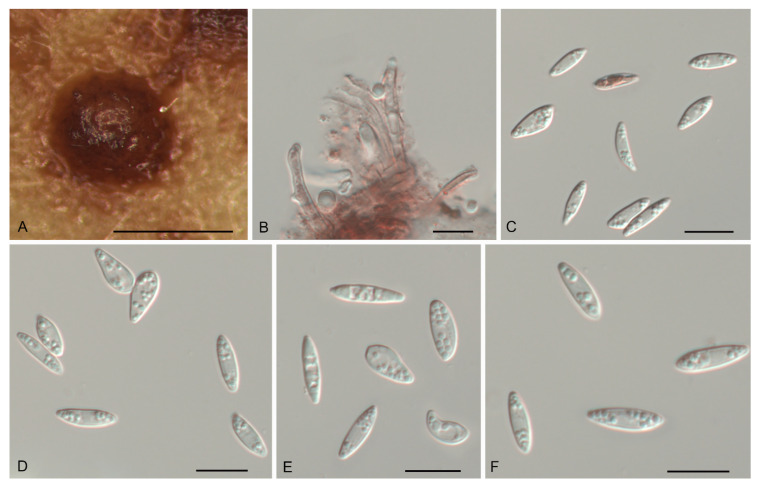
Morphology of *Gnomoniopsis fagacearum* (CFCC 54316). (**A**) Conidioma formed on PDA; (**B**) Conidiogenous cells giving rise to conidia; (**C**–**F**) Conidia. Scale bars: A = 300 μm; (**B**–**F**) = 10 μm.

*Gnomoniopsis fagacearum* N. Jiang, sp. nov. Figure 5.

Mycobank No.: 840970.

Etymology—Named after the host family, *Fagaceae*.

Description—*Conidiomata* acervular, solitary, erumpent, pulvinate, red-brown, 250–450 μm diam. *Conidiophores* indistinct, often reduced to conidiogenous cells. *Conidiogenous cells* red-brown, smooth, multi-guttulate, cylindrical, slightly curved, attenuate towards apex, phialidic, 16–33.5 × 2–5 μm. *Conidia* aseptate, hyaline or seldom red-brown, smooth, multi-guttulate, fusoid, straight or curved, base truncate, (9–) 9.6–11.4 (–12.6) × (2.8–) 3.1–4 (–4.5) μm (*n* = 50), L/W = 2.1–4.2.

Culture characteristics—Colonies flat, spreading, with moderate aerial mycelium, folded surface and lobate margin, sienna to red-brown on MEA, dirty-white to slightly red-brown on PDA, occasionally forming red-brown conidiomata.

Material examined—CHINA, Guangdong Province, Qingyuan City, Yangshan County, on diseased leaves of *Lithocarpus glaber*, 26 November 2019, Dan-Ran Bian (JNH0005 *holotype*; *ex-type living culture*, CFCC 54316); Jiangxi Province, Xinyu City, Fenyi County, on diseased leaves of *Castanopsis faberi*, 20 October 2019, Yong Li (living culture, CFCC 54288); Shaanxi Province, Hanzhong City, Foping County, on diseased leaves of *Quercus variabilis*, 13 August 2019, Yong Li (living culture, CFCC 54439); Fujian Province, Nanping City, Yanping County, on diseased leaves of *Castanopsis eyrei*, 13 July 2019, Dan-Ran Bian (living culture, CFCC 54414); Guangdong Province, Qingyuan City, Yangshan County, on diseased leaves of *Castanopsis chunii*, 26 November 2019, Dan-Ran Bian (living culture, CFCC 54412).

Notes—Five isolates from leaf spots of *Castanopsis chunii*, *C. eryei*, *C. faberi*, *Lithocarpus glaber* and *Quercus variabilis* clustered into a well-supported clade here newly described as *Gnomoniopsis fagacearum*, which is distinct from any known species phylogenetically (Figure 1 and Figure 2). Morphologically, *G. guangdongensis* can be distinguished from the other *Gnomoniopsis* species by red-brown conidiogenous cells.

**Figure 6:**
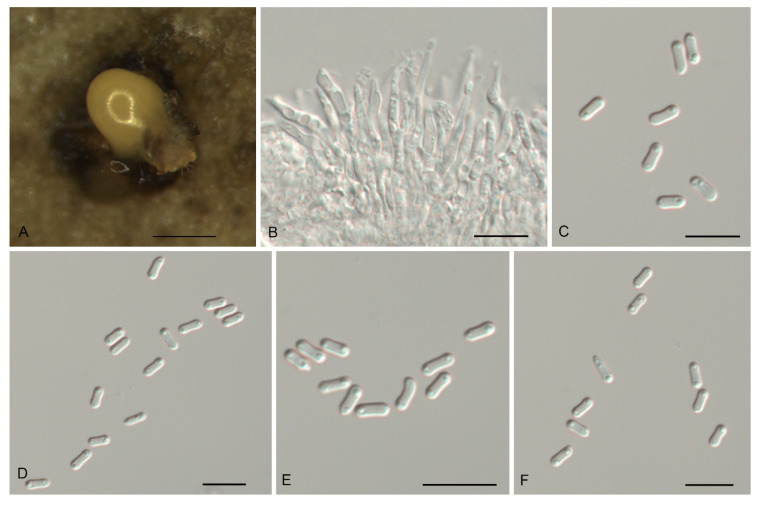
Morphology of *Gnomoniopsis guangdongensis* (CFCC 54443). (**A**) Conidioma formed on PDA; (**B**) Conidiogenous cells giving rise to conidia; (**C**–**F**) Conidia. Scale bars: A = 300 μm; (**B**–**F**) = 10 μm.

*Gnomoniopsis guangdongensis* N. Jiang, sp. nov. Figure 6.

Mycobank No.: 840971.

Etymology—Named after the collection site, Guangdong Province.

Description—*Conidiomata* pycnidial, aggregated or solitary, erumpent, globose to pulvinate, dark brown, 150–600 μm diam., exuding a creamy conidial mass. *Conidiophores* indistinct, often reduced to conidiogenous cells. *Conidiogenous cells* hyaline, smooth, multi-guttulate, cylindrical to ampulliform, attenuate towards apex, phialidic, 12.5–24 × 1.5–3 μm. *Conidia* aseptate, hyaline, smooth, multi-guttulate, cylindrical, constricted at the middle, straight or slightly curved, base truncate, (4.3–) 4.6–5 (–5.2) × (1.4–) 1.6–1.8 (–2) μm (*n* = 50), L/W = 2.4–3.3.

Culture characteristics—Colonies flat, spreading, with sparse to moderate aerial mycelium and diffuse margin, buff to fawn on MEA, dirty-white on PDA, with age forming narrow concentric zones, forming abundant dark brown conidiomata with creamy conidial masses.

**Material examined**—CHINA, Guangdong Province, Qingyuan City, Yangshan County, on diseased leaves of *Castanopsis fargesii*, 26 November 2019, Dan-Ran Bian (JNH0006 *holotype*; *ex-type living culture*, CFCC 54443); *Ibid*. (living cultures CFCC 54331 and CFCC 54282).

Notes—Three isolates from leaf spots of *Castanopsis fargesii* clustered into a well-supported clade named *Gnomoniopsis guangdongensis*, which is distinct from any known species phylogenetically (Figure 1 and Figure 2). Morphologically, *G. guangdongensis* can be distinguished from the other *Gnomoniopsis* species by its conidia constricted at the middle.

**Figure 7:**
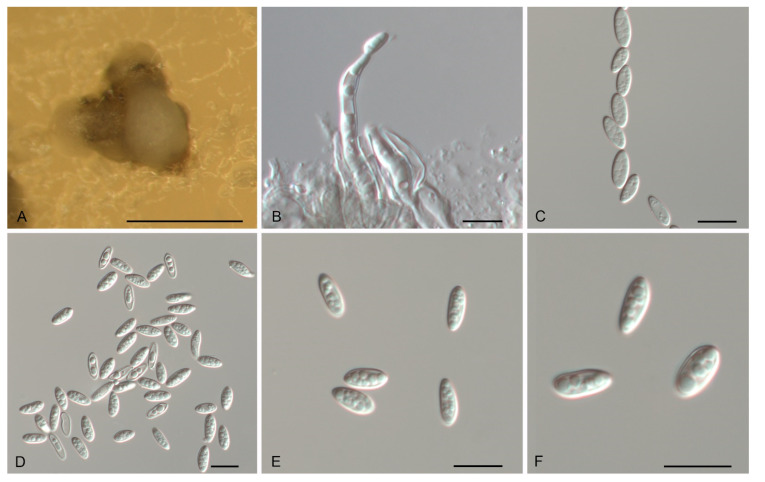
Morphology of *Gnomoniopsis hainanensis* (CFCC 54376). (**A**) Conidioma formed on PDA; (**B**) Conidiogenous cells giving rise to conidia; (**C**–**F**) Conidia. Scale bars: A = 300 μm; (**B**–**F**) = 10 μm.

*Gnomoniopsis hainanensis* N. Jiang, sp. nov. Figure 7.

Mycobank No.: 840972.

Etymology—Named after the collection site, Hainan Province.

Description—*Conidiomata* pycnidial, solitary, erumpent, globose to pulvinate, light brown, 100–300 μm diam., exuding a creamy conidial mass. *Conidiophores* indistinct, often reduced to conidiogenous cells. *Conidiogenous cells* hyaline, smooth, multi-guttulate, cylindrical, attenuate towards apex, phialidic, 16.5–26 × 2.5–4.5 μm. *Conidia* aseptate, hyaline, smooth, multi-guttulate, fusoid, straight, base truncate, (7.3–) 8–10 (–12.2) × (3.3–) 3.4–3.9 (–4.2) μm (*n* = 50), L/W = 1.9–3.3.

Culture characteristics—Colonies flat, spreading, with sparse aerial mycelium and lobate to undulate margin, sienna to luteous on MEA, luteous on PDA, with age forming narrow concentric zones, forming abundant light brown conidiomata with creamy conidial masses.

Material examined—CHINA, Hainan Province, Changjiang Li Autonomous County, on diseased leaves of *Castanopsis hainanensis*, 16 November 2018, Yong Li (JNH0007 *holotype*; *ex-type living culture*, CFCC 54376); *Ibid*. (living culture CFCC 55877).

Notes—Two isolates from leaf spots of *Castanopsis hainanensis* clustered into a well-supported clade here newly described as *Gnomoniopsis hainanensis*, which is distinct from any known species phylogenetically (Figure 1 and Figure 2). *G. guangdongensis* is different from the phylogenetically close species *G. fagacearum* by its conidial size and length-width ratio (7.3–12.2 × 3.3–4.2 μm, L/W = 1.9–3.3 in *G. guangdongensis* vs. 9–12.6 × 2.8–4.5 μm, L/W = 2.1–4.2 in *G. fagacearum*).

**Figure 8:**
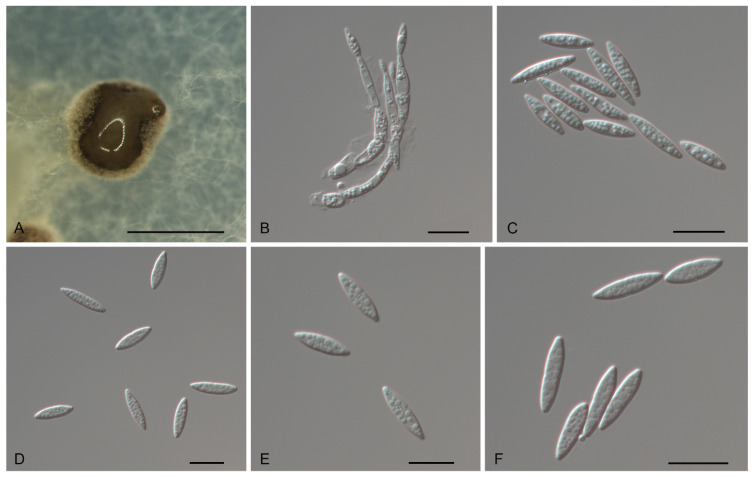
Morphology of *Gnomoniopsis rossmaniae* (CFCC 54307). (**A**) Conidioma formed on PDA; (**B**) Conidiogenous cells giving rise to conidia; (**C**–**F**) Conidia. Scale bars: A = 300 μm; (**B**–**F**) = 10 μm.

*Gnomoniopsis rossmaniae* N. Jiang, sp. nov. Figure 8.

Mycobank No.: 840973.

Etymology—In honor of Amy Y. Rossman for her contributions to the study of the fungal order *Diaporthales*.

Description—*Conidiomata* pycnidial, solitary, erumpent, pulvinate, dark brown, 250–650 μm diam., exuding a brown conidial mass. *Conidiophores* indistinct, often reduced to conidiogenous cells. *Conidiogenous cells* hyaline, smooth, multi-guttulate, cylindrical to ampulliform, attenuate towards apex, phialidic, 9–19 × 2–3 μm. *Conidia* aseptate to 1-septate, slightly constricted at septum, hyaline, smooth, multi-guttulate, elongate-fusoid, straight, base truncate, (10–) 11.6–14.6 (–16.1) × (3.1–) 3.3–3.9 (–4.1) μm (*n* = 50), L/W = 2.8–4.5.

Culture characteristics—Colonies flat, spreading, with sparse aerial mycelium and lobate to undulate margin, hazel on MEA, dirty-white on PDA, seldom forming dark brown conidiomata with brown conidial masses.

Material examined—CHINA, Hainan Province, Changjiang Li Autonomous County, on diseased leaves of *Castanopsis hainanensis*, 16 November 2018, Yong Li (JNH0008 *holotype*; *ex-type living culture*, CFCC 54307); *Ibid*. (living culture CFCC 55876).

Notes—Two isolates from leaf spots of *Castanopsis hainanensis* clustered into a well-supported clade here newly described as *Gnomoniopsis rossmaniae*, which is distinct from any known species phylogenetically (Figure 1 and Figure 2). Morphologically, *G. rossmaniae* can be distinguished from the other *Gnomoniopsis* species by its aseptate to 1-septate, elongate-fusoid conidia.

**Figure 9:**
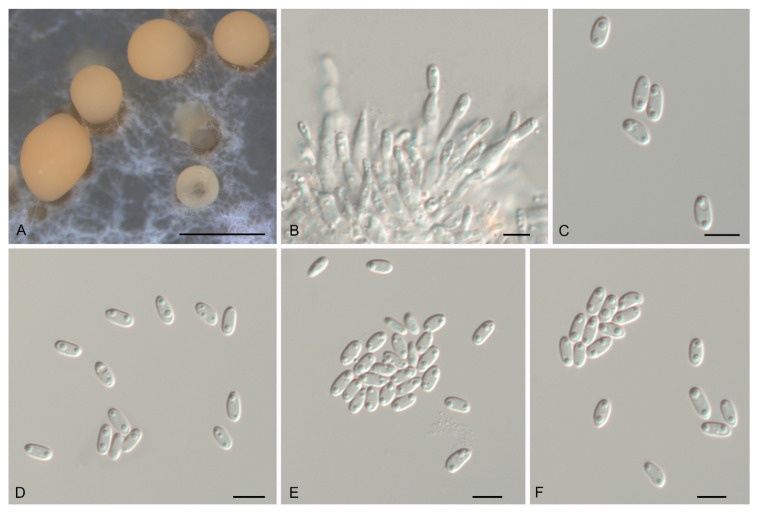
Morphology of *Gnomoniopsis silvicola* (CFCC 54418). (**A**) Conidiomata formed on PDA; (**B**) Conidiogenous cells giving rise to conidia; (**C**–**F**) Conidia. Scale bars: A = 500 μm; (**B**–**F**) = 5 μm.

*Gnomoniopsis silvicola* N. Jiang, sp. nov. Figure 9.

Mycobank No.: 840974.

Etymology—Name from “silva” = forest and “-cola” = inhabiting; with reference to its woody host.

Description—*Conidiomata* pycnidial, aggregated or solitary, erumpent, globose to pulvinate, brown, 250–650 μm diam., exuding a creamy conidial mass. *Conidiophores* indistinct, often reduced to conidiogenous cells. *Conidiogenous cells* hyaline, smooth, multi-guttulate, cylindrical to ampulliform, attenuate towards apex, phialidic, 7–15 × 1.5–2.5 μm. *Conidia* aseptate, hyaline, smooth, multi-guttulate, oval to fusoid, straight or slightly curved, base truncate, (4.3–) 4.5–5.3 (–5.9) × (1.9–) 2.2–2.6 (–2.7) μm (*n* = 50), L/W = 1.7–2.5.

Culture characteristics—Colonies flat, spreading, with moderate aerial mycelium and undulate margin, luteous to brown on MEA, dirty-white on PDA, forming abundant brown conidiomata with creamy conidial masses.

*Material examined*—CHINA, Shaanxi Province, Hanzhong City, Foping County, on diseased leaves of *Quercus serrata*, 13 August 2019, Yong Li (JNH0009 *holotype*; *ex-type living culture*, CFCC 54418); Guangdong Province, Shaoguan City, Lechang County, on diseased leaves of *Castanopsis hystrix*, 4 December 2019, Dan-Ran Bian (living culture, CFCC 54304).

Notes—Two isolates from leaf spots of *Castanopsis hystrix* and *Quercus serrata* clustered into a well-supported clade here described as the new species *Gnomoniopsis silvicola*, which is distinct from any known species phylogenetically (Figure 1 and Figure 2). Morphologically, *G. silvicola* has a bit smaller conidia than its phylogenetically close species *G. daii* (4.3–5.9 × 1.9–2.7 μm in *G. silvicola* vs. 5.1–6.3 × 2.3–3.6 μm in *G. daii*). In addition, *G. silvicola* is separated from *G. daii* in 34 bp differences in ITS.

## 4. Discussion

**Figure 10:**
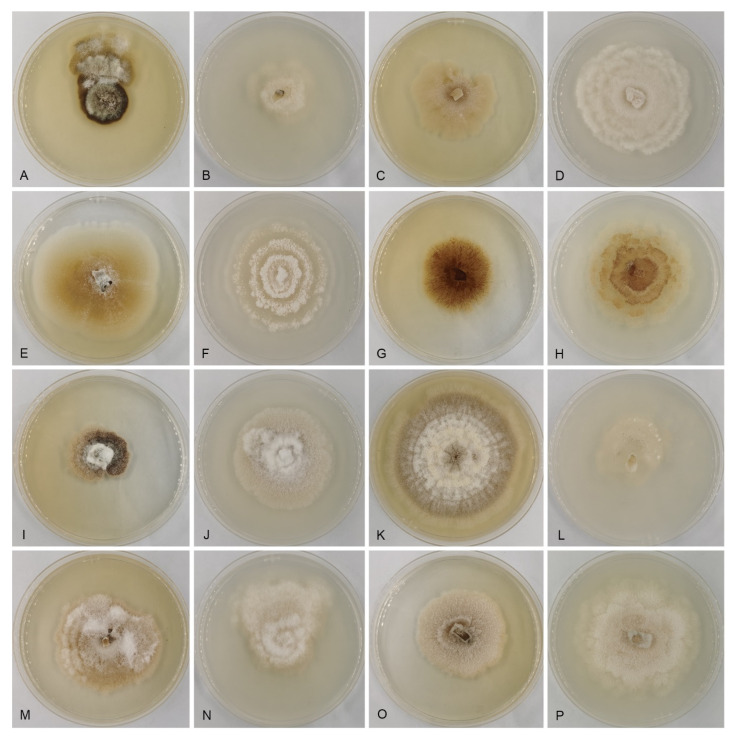
*Gnomoniopsis* cultures at 10 days. (**A**) *G. silvicola* (CFCC 54304) on MEA; (**B**) *G. silvicola* (CFCC 54304) on PDA; (**C**) *G. rossmaniae* (CFCC 54307) on MEA; (**D**) *G. rossmaniae* (CFCC 54307) on PDA; (**E**) *G. guangdongensis* (CFCC 54443) on MEA; (**F**) *G. guangdongensis* (CFCC 54443) on PDA; (**G**) *G. hainanensis* (CFCC 54376) on MEA; (**H**) *G. hainanensis* (CFCC 54376) on PDA; (**I**) *G. fagaceaerum* (CFCC 54316) on MEA; (**J**) *G. fagaceaerum* (CFCC 54316) on PDA; (**K**) *G. silvicola* (CFCC 54418) on MEA; (**L**) *G. silvicola* (CFCC 54418) on PDA; (**M**) *G. castanopsidis* (CFCC 54437) on MEA; (**N**) *G. castanopsidis* (CFCC 54437) on PDA; (**O**) *G. daii* (CFCC 55517) on MEA; (**P**) *G. daii* (CFCC 55517) on PDA.

In the present study, six new *Gnomoniopsis* species (viz. *G. castanopsidis*, *G. fagacearum*, *G*. *guangdongensis*, *G*. *hainanensis*, *G*. *rossmaniae* and *G*. *silvicola*) are described and illustrated (Figure 3, Figure 4, Figure 5, Figure 6, Figure 7, Figure 8, Figure 9 and Figure 10), and a new host, *Quercus aliena*, is reported for the known species *G. daii*. As noted in previous studies, the fungal genus *Gnomoniopsis* is so far only known from hosts of three plant families, *Fagaceae*, *Onagraceae* and *Rosaceae* [20,21,40], of which only one species, *G. racemula* was described from the family *Onagraceae* [20]. Hence, *Fagaceae* and *Rosaceae* are the main hosts for *Gnomoniopsis* species. Although several new species and host records are reported from *Fagaceae* in China herein, numerous additional hidden species might remain to be revealed from the widely spread fagaceous species in China.

So far, eleven *Gnomoniopsis* species were reported from fagaceous hosts, of which *G. clavulata* and *G. paraclavulata* were described from *Quercus* in the USA [20]. *Gnomoniopsis smithogilvyi* was reported as causal agent of sweet chestnut fruit rot in Australia, Europe and North America [25,26,41,42,43,44,45]. The remaining eight species are only known from China. They were well distinguished in phylogenetic analyses of the ITS gene and of combined matrices of ITS, *tef1* and *tub2* genes (Figure 1 and Figure 2). The conidial characters as well as the hosts and distribution provide useful information for species delimitation (Table 2).

Several *Gnomoniopsis* species are pathogens of leaves, branches or fruits [29,46]. For example, *G. smithogilvyi* causes sweet chestnut branch canker and fruit rot in in Australia, Europe and the USA [26,42,45], whereas in China *G. daii* is one of the main pathogens of Chinese chestnut causing fruit rot and leaf spot diseases [23,28]. In addition, *G. chinensis* causes branch canker of Chinese chestnut in China [29]. The newly described species of the present study were isolated from diseased leaves; however, additional studies are required to confirm their pathogenicity.

## 5. Conclusions

Eight *Gnomoniopsis* species are known from fagaceous hosts in China based on morphology and phylogeny, viz. *G. chinensis* on *Castanea mollissima*, *G. castanopsidis* on *Castanopsis hystrix*, *G. daii* on *Castanea mollissima* and *Quercus aliena*, *G. fagacearum* on *Castanopsis chunii*, *Castanopsis eyrei*, *Castanopsis faberi*, *Lithocarpus glaber* and *Quercus variabilis*, *G. guangdongensis* on *Castanopsis fargesii*, *G. hainanensis* on *Castanopsis hainanensis*, *G. rossmaniae* on *Castanopsis hainanensis* and *G. silvicola* on *Castanopsis hystrix* and *Quercus serrata*. They can be well distinguished by the combined approaches of morphology and phylogeny based on ITS, *tef1* and *tub2* genes.

## Figures and Tables

**Table 1:** Strains and GenBank accession numbers used in this study.

Species	Country	Host	Host Family	Strain	GenBank Accession Number		
					ITS	*tef1*	*tub2*
*Apiognomonia errabunda*	Switzerland	*Fagus sylvatica*	*Fagaceae*	AR 2813	DQ313525	DQ313565	DQ862014
*Gnomoniopsis alderdunensis*	USA	*Rubus pedatus*	*Rosaeace*	CBS 125679	GU320826	GU320813	GU320788
*Gnomoniopsis alderdunensis*	USA	*Rubus parviflorus*	*Rosaeace*	CBS 125680 *	GU320825	GU320801	GU320787
*Gnomoniopsis alderdunensis*	USA	*Rubus parviflorus*	*Rosaeace*	CBS 125681	GU320827	GU320802	GU320789
*Gnomoniopsis chamaemori*	Finland	*Rubus chamaemorus*	*Rosaeace*	CBS 804.79	GU320817	GU320809	GU320777
*Gnomoniopsis chinensis*	China	*Castanea mollissima*	*Fagaceae*	CFCC 52286 *	MG866032	MH545370	MH545366
*Gnomoniopsis chinensis*	China	*Castanea mollissima*	*Fagaceae*	CFCC 52287	MG866033	MH545371	MH545367
*Gnomoniopsis chinensis*	China	*Castanea mollissima*	*Fagaceae*	CFCC 52288	MG866034	MH545372	MH545368
*Gnomoniopsis chinensis*	China	*Castanea mollissima*	*Fagaceae*	CFCC 52289	MG866035	MH545373	MH545369
*Gnomoniopsis clavulata*	USA	*Quercus falcata*	*Fagaceae*	CBS 121255	EU254818	GU320807	EU219211
***Gnomoniopsis castanopsidis***	**China**	***Castanopsis hystrix***	***Fagaceae***	CFCC 54437 *	**MZ902909**	**MZ936385**	**NA**
***Gnomoniopsis castanopsidis***	**China**	***Castanopsis hystrix***	***Fagaceae***	**CFCC 55878**	**MZ902910**	**MZ936386**	**NA**
*Gnomoniopsis comari*	Finland	*Comarum palustre*	*Rosaeace*	CBS 806.79	EU254821	GU320810	EU219156
*Gnomoniopsis comari*	Finland	*Comarum palustre*	*Rosaeace*	CBS 807.79	EU254822	GU320814	GU320779
*Gnomoniopsis comari*	Switzerland	*Comarum palustre*	*Rosaeace*	CBS 809.79	EU254823	GU320794	GU320778
*Gnomoniopsis daii*	China	*Castanea mollissima*	*Fagaceae*	CFCC 54043 *	MN598671	MN605517	MN605519
*Gnomoniopsis daii*	China	*Castanea mollissima*	*Fagaceae*	CMF002B	MN598672	MN605518	MN605520
***Gnomoniopsis daii***	**China**	***Quercus aliena***	***Fagaceae***	**CFCC 55517**	**MZ902911**	**MZ936387**	**MZ936403**
***Gnomoniopsis daii***	**China**	***Quercus aliena***	***Fagaceae***	**CFCC 55294B**	**MZ902912**	**MZ936388**	**MZ936404**
***Gnomoniopsis fagacearum***	**China**	***Castanopsis faberi***	***Fagaceae***	**CFCC 54288**	**MZ902913**	**MZ936389**	**MZ936405**
***Gnomoniopsis fagacearum***	**China**	***Quercus variabilis***	***Fagaceae***	**CFCC 54439**	**MZ902914**	**MZ936390**	**MZ936406**
***Gnomoniopsis fagacearum***	**China**	***Castanopsis eyrei***	***Fagaceae***	**CFCC 54414**	**MZ902915**	**MZ936391**	**MZ936407**
***Gnomoniopsis fagacearum***	**China**	***Lithocarpus glaber***	***Fagaceae***	CFCC 54316 *	**MZ902916**	**MZ936392**	**MZ936408**
***Gnomoniopsis fagacearum***	**China**	***Castanopsis chunii***	***Fagaceae***	**CFCC 54412**	**MZ902917**	**MZ936393**	**MZ936409**
*Gnomoniopsis fragariae = G. fructicola*	USA	*Fragaria vesca*	*Rosaeace*	CBS 121226	EU254824	GU320792	EU219144
*Gnomoniopsis fragariae = G. fructicola*	France	*Fragaria* sp.	*Rosaeace*	CBS 208.34	EU254826	GU320808	EU219149
*Gnomoniopsis fragariae = G. fructicola*	USA	*Fragaria* sp.	*Rosaeace*	CBS 125671	GU320816	GU320793	GU320776
***Gnomoniopsis guangdongensis***	**China**	***Castanopsis fargesii***	***Fagaceae***	CFCC 54443 *	**MZ902918**	**MZ936394**	**MZ936410**
***Gnomoniopsis guangdongensis***	**China**	***Castanopsis fargesii***	***Fagaceae***	**CFCC 54331**	**MZ902919**	**MZ936395**	**MZ936411**
***Gnomoniopsis guangdongensis***	**China**	***Castanopsis fargesii***	***Fagaceae***	**CFCC 54282**	**MZ902920**	**MZ936396**	**MZ936412**
*Gnomoniopsis guttulata*	Bulgaria	*Agrimonia eupatoria*	*Rosaeace*	MS 0312	EU254812	NA	NA
***Gnomoniopsis hainanensis***	**China**	***Castanopsis hainanensis***	***Fagaceae***	CFCC 54376 *	**MZ902921**	**MZ936397**	**MZ936413**
***Gnomoniopsis hainanensis***	**China**	***Castanopsis hainanensis***	***Fagaceae***	**CFCC 55877**	**MZ902922**	**MZ936398**	**MZ936414**
*Gnomoniopsis idaeicola*	USA	*Rubus* sp.	*Rosaeace*	CBS 125672	GU320823	GU320797	GU320781
*Gnomoniopsis idaeicola*	USA	*Rubus pedatus*	*Rosaeace*	CBS 125673	GU320824	GU320798	GU320782
*Gnomoniopsis idaeicola*	France	*Rubus* sp.	*Rosaeace*	CBS 125674	GU320820	GU320796	GU320780
*Gnomoniopsis idaeicola*	USA	*Rubus procerus*	*Rosaeace*	CBS 125675	GU320822	GU320799	GU320783
*Gnomoniopsis idaeicola*	USA	*Rubus procerus*	*Rosaeace*	CBS 125676	GU320821	GU320811	GU320784
*Gnomoniopsis macounii*	USA	*Spiraea* sp.	*Rosaeace*	CBS 121468	EU254762	GU320804	EU219126
*Gnomoniopsis occulta*	USA	*Potentilla* sp.	*Rosaeace*	CBS 125677	GU320828	GU320812	GU320785
*Gnomoniopsis occulta*	USA	*Potentilla* sp.	*Rosaeace*	CBS 125678	GU320829	GU320800	GU320786
*Gnomoniopsis paraclavulata*	USA	*Quercus alba*	*Fagaceae*	CBS 123202	GU320830	GU320815	GU320775
*Gnomoniopsis racemula*	USA	*Chamerion angustifolium*	*Onagraceae*	CBS 121469 *	EU254841	GU320803	EU219125
***Gnomoniopsis rossmaniae***	**China**	***Castanopsis hainanensis***	***Fagaceae***	CFCC 54307 *	**MZ902923**	**MZ936399**	**MZ936415**
***Gnomoniopsis rossmaniae***	**China**	***Castanopsis hainanensis***	***Fagaceae***	**CFCC 55876**	**MZ902924**	**MZ936400**	**MZ936416**
*Gnomoniopsis sanguisorbae*	Switzerland	*Sanguisorba minor*	*Rosaeace*	CBS 858.79	GU320818	GU320805	GU320790
***Gnomoniopsis silvicola***	**China**	***Castanopsis hystrix***	***Fagaceae***	**CFCC 54304**	**MZ902925**	**MZ936401**	**MZ936417**
***Gnomoniopsis silvicola***	**China**	***Quercus serrata***	***Fagaceae***	CFCC 54418 *	**MZ902926**	**MZ936402**	**MZ936418**
*Gnomoniopsis smithogilvyi*	Australia	*Castanea* sp.	*Fagaceae*	CBS 130190 *	JQ910642	KR072534	JQ910639
*Gnomoniopsis smithogilvyi*	Australia	*Castanea* sp.	*Fagaceae*	CBS 130189	JQ910644	KR072535	JQ910641
*Gnomoniopsis smithogilvyi*	Australia	*Castanea* sp.	*Fagaceae*	CBS 130188	JQ910643	KR072536	JQ910640
*Gnomoniopsis smithogilvyi*	Italy	*Castanea sativa*	*Fagaceae*	MUT 401	HM142946	KR072537	KR072532
*Gnomoniopsis smithogilvyi*	New Zealand	*Castanea sativa*	*Fagaceae*	MUT 411	HM142948	KR072538	KR072533
*Gnomoniopsis tormentillae*	Switzerland	*Potentilla* sp.	*Rosaeace*	CBS 904.79	EU254856	GU320795	EU219165
*Gnomoniopsis xunwuensis*	China	*Castanopsis fissa*	*Fagaceae*	CFCC 53115 *	MK432667	MK578141	MK578067
*Gnomoniopsis xunwuensis*	China	*Castanopsis fissa*	*Fagaceae*	CFCC 53116	MK432668	MK578142	MK578068

Note: NA, not applicable. Ex-type strains are marked with *, and strains from present study are in black bold.

**Table 2:** Comparison of *Gnomoniopsis* species on hosts belonging to *Fagaceae*.

Species	Host	Conidial Length (µm)	Conidial Width (µm)	L/W Ratio	Reference
*G. castanopsidis*	*Castanopsis hystrix*	(4.3–) 4.6–5.1 (–5.4)	(1.8–) 2.1–2.5 (–2.6)	1.8–2.6	This study
*G. chinensis*	*Castanea mollissima*	(6.0–) 6.5–8.5 (–9.0)	(2.2–) 2.7–3 (–3.5)	NA	[29]
*G. clavulata*	*Quercus falcata*	(5–) 6–6.5 (–8)	(2–) 2.5–3 (–4)	1.4–3.7	[20]
*G. daii*	*Castanea mollissima*	(5.0–) 5.5–7.0 (–8.0)	2.0–3.5	NA	[23,28]
*G. daii*	*Quercus aliena*	(5.1–) 5.6–6.1 (–6.3)	(2.3–) 2.8–3.2 (–3.6)	1.4–2.5	This study
*G. fagacearum*	*Castanopsis chunii*, *C. eryei*, *C. faberi*, *Lithocarpus glaber* and *Quercus variabilis*	(9–) 9.6–11.4 (–12.6)	(2.8–) 3.1–4 (–4.5)	2.1–4.2	This study
*G. guangdongensis*	*Castanopsis fargesii*	(4.3–) 4.6–5 (–5.2)	(1.4–) 1.6–1.8 (–2)	2.4–3.3	This study
*G. hainanensis*	*Castanopsis hainanensis*	(7.3–) 8–10 (–12.2)	(3.3–) 3.4–3.9 (–4.2)	1.9–3.3	This study
*G. paraclavulata*	*Quercus alba*	(6–) 7.5–8 (–9.5)	(2–) 3–3 (–3.5)	1.6–4.2	[20]
*G. rossmaniae*	*Castanopsis hainanensis*	(10–) 11.6–14.6 (–16.1)	(3.1–) 3.3–3.9 (–4.1)	2.8–4.5	This study
*G. silvicola*	*Castanopsis hystrix* and *Quercus serrata*	(4.3–) 4.5–5.3 (–5.9)	(1.9–) 2.2–2.6 (–2.7)	1.7–2.5	This study
*G. smithogilvyi*	*Castanea sativa*	(6.0–) 8 (–9.5)	(2.0–) 2.5 (–4.0)	2.5–3.5	[25]

## Data Availability

The sequences from the present study were submitted to the NCBI website (https://www.ncbi.nlm.nih.gov/) and the accession numbers were listed in Table 1.
